# Expression of Granulisyn, Perforin and Granzymes in Human Milk over Lactation and in the Case of Maternal Infection

**DOI:** 10.3390/nu10091230

**Published:** 2018-09-04

**Authors:** Alecia-Jane Twigger, Gwendoline K. Küffer, Donna T. Geddes, Luis Filgueria

**Affiliations:** 1 Institute for Stem Cell Research, Helmholtz Center Munich, 85764 Munich, Germany; 2 Faculty of Science and Medicine, University of Fribourg, 1700 Fribourg, Switzerland; gwendoline.kueffer@gmail.com (G.K.K.); luis.filgueira@unifr.ch (L.F.); 3 School of Molecular Sciences, Faculty of Science, The University of Western Australia, Perth 6009, Australia; donna.geddes@uwa.edu.au

**Keywords:** human milk, milk cells, immune cells, antimicrobial proteins

## Abstract

Human milk has been previously found to contain various types of leukocytes however specific characteristics of these cells, such as whether they contain cytolytic antimicrobial proteins that may induce pathogen directed cell death, are unknown. This project aims to examine the presence and localization of immune proteins such as perforin, granulysin and granzymes in human milk cells at the protein and mRNA level. Genes encoding these proteins were confirmed in human milk cell samples, which were particularly enriched in early milk and in the case of maternal infection. Fluorescence activated cell sorting (FACS) was used to investigate the co-expression of these proteins with pan-immune cell marker CD45 and epithelial marker EPCAM. Co-expression of antimicrobial proteins was found predominantly in CD45 positive cells, also increasing in the case of maternal infection. Our study suggests that human milk contains cells that carry hallmarks of activated or memory T-cells which are enriched early in lactation and in the case of maternal infection. Presence and prevalence of these cells in human milk may indicate a role in the protection of the maternal breast or for delivery to the vulnerable infant.

## 1. Introduction

Human milk contains all the necessary components required to satisfy the nutritional requirements of the infant, as well as immunological factors that support survival and thriving of the child. At birth, full term newborns are exposed to both vaginal bacteria and gut microbiota, some of which may be pathogenic [1]. Whilst infants have more or less a complete immune system, it is still highly immature [1,2,3] and requires contact with foreign antigens and stimulation such as from human milk to develop effective and specific defence mechanisms [2,3]. Human milk proteins, such as lactoferrin, certain caseins [4,5] and lysozyme, along with other bioactive molecules such as oligosaccharides, defensins, cytokines, chemokines, growth factors and anti-oxidants [1,3] support the development of the immune system at the same time as providing antimicrobial and antiviral effects [2]. Of note, secretory IgA (sIgA) antibodies found in human milk and produced by maternal plasma cells is an important antibody directed against microbes and food antigens that have challenged the maternal immune response [2]. The presence of immune cells, such as lymphocytes, has been shown in human milk [1,2,3,6,7], which increase in number when the infant has an infection even in an asymptomatic mother [8]. Moreover, the immune cell content of milk increases to a much greater degree during maternal infection, particularly during mastitis. It is therefore hypothesized that leukocytes entering human milk might play a role for the infant but may also result from protection of the breast tissue [3].

Generally, if a pathogenic or non-host cell (such as bacteria) is detected, the immune system is activated, leading primed leukocytes such as cytotoxic t-cells and natural killer cells to release cytolytic granules. These granules contain the bioactive molecules perforin [9] and granulysin (synthetic drugs include novobiocin [10]), which work together to form pores in the cell to allow the entry of a third component, granzymes into pathogenic cells, and trigger bacterial cell programmed death and possibly also apoptosis of the host cell [11,12,13,14]. Granzymes are serine proteases and of the twelve granzymes already described, five have been found in the human (A, B, H, K and M) and ten have been identified in rodents (A-G, K, M and N) [13]. Whilst immune cells are known to exist in human milk with their numbers and distribution changing in relation to the health status of either the mother or the child [3,7,8,15], the presence of associated cytotoxic immune proteins perforin, granzymes and granulysin have not yet been identified in human milk cells. Presence of these proteins in human milk leukocytes would indicate the existence of activated or memory t-cells which have recently or may be actively fighting pathogenic cells which may be of importance either for the protection of the maternal breast or infant. The aim of this study is to determine the presence of these antimicrobial proteins in human milk cells and determine whether they are normally expressed across different stages of lactation in healthy participants or in the case of breast inflammatory conditions such as mastitis.

## 2. Materials and Methods

### 2.1. Human Milk Collection

The study was approved by the Swissethics Committee (2016-00309, Switzerland), the Human Research Ethics Committee of The University of Western Australia (UWA, RA/4/1/4397) and the Australian Breastfeeding Association (ABA; 2014–5). All participants provided informed written consent to engage in the study and all methods were carried out according to the approved guidelines. Thirty-six mothers were recruited through the Hospital of Fribourg, ABA meetings and the website humanlactationresearchgroup.com. Mothers were on average 34.4 years of age (range from 27 to 45 years of age) (Table 1). All infants, with 53% being male, were born term with a median of 278 days of gestation (range from 249 to 301 days) with 60% delivered vaginally (Table 1). Milk samples were collected 4–142 days post-partum and a single pre-partum secretion was collected 5 days before birth (Table 1). After collection, milk samples were brought directly to the laboratory to be processed.

### 2.2. Milk Cell Isolation

Each milk sample was diluted in equal volume of Phosphate Buffer Saline (PBS) (Gibco, Thermo Fisher Scientific, Wilmington, DE, USA) and centrifuged at 800 g for 20 min at 20 °C. The fat and skim layer of the milk was removed before washing the cell pellet twice in sterile PBS and the cells were resuspended in 5–10 mL of PBS. Cells were used fresh for flow cytometry or frozen and stored at −80 °C for RNA extraction and corresponding analysis.

### 2.3. RNA Extraction

Total RNA was extracted from frozen cell pellets, previously collected as part of a larger study. Mini RNeasy extraction kit (Qiagen, Valencia, CA, USA) was used according to the manufacturer’s protocol. The concentration and purity of RNA was measured using NanoDrop 2000 (Thermo Fisher Scientific, Wilmington, DE, USA). All extracted RNA was of a high quality with a 260/280 ratio between 1.8 and 2.2. Pooled resting mammary tissue RNA taken from five donors aged 40–55 was purchased from Aligent Technologies (Catalogue number: 540045, lot number: 0006135096, Aligent Technologies, Santa Clara, CA, USA).

### 2.4. cDNA Generation

RNA was reverse transcribed into cDNA using the cDNA archive kit (Life Technologies, Carlsbad, CA, USA) following the manufacturer’s instructions. 50 μL reactions were incubated in a Bio-Rad C1000 96-well gradient block thermal cycler and held at 25 °C for 10 min, followed by 37 °C for 120 min, 85 °C for 5 min and finally at 4 °C until collection.

### 2.5. Quantitative Real Time Polymerase Chain Reaction (qRT-PCR)

Gene expression was investigated through quantitative real time PCR using Taqman probes (Table S1, Life Technologies, Thermo fisher, CA, USA) with the 7500 Fast qRT-PCR system (Life Technologies). Each sample was measured in triplicate or where necessary, in duplicate. Cycle time (CT) values were obtained for each sample and subsequently, relative quantitation (RQ) was calculated using 2^Ct(control)-Ct(sample)^ ± SD, where genes were normalized to resting breast tissue and GAPDH was used as a housekeeping control gene. 

### 2.6. Sequencing Library Research

Genes coding for cytolytic immune proteins perforin (PRF1), granulysin (GNLY) and granzymes A, B, H and M (GZMA, GZMB, GZMH, GZMM) were searched in an RNA-sequencing dataset [16], which explored the transcriptome of prepartum secretions (PS) and human milk (HM) cells as well as resting mammary tissue (RMT). Previously, 1.1 × 10^5^ − 19.3 × 10^5^ cells/mL were isolated from PS samples collected from four women at 38–40 weeks of pregnancy. All participants provided follow-up samples of 0.4 × 10^6^ − 43.5 × 10^6^ cells/mL HM at 1, 3, 6 and 12 months of lactation [16]. mRNA was extracted from the isolated cells, the quantity was then standardized [17,18] and the samples were processed for library preparation. Moreover, RMT taken from five women aged 40−55 years (Catalogue number: 540045, Lot number: 0006135096, Agilent Technologies, Santa Clara, CA, USA) was pooled and mRNA was likewise processed for library preparation. Illumina HiSeq2500 version 3 was used to sequence all samples with a production of a minimum of 20 million 50 base paired single end reads. SOAP aligner 2 was used to align 865,913,217 clean reads to the human genome where only 2 mismatches were allowed, resulting in 414,203,980 clean transcripts. Gene expression levels were expressed as RPKM (Reads Per Kilobase per Million mapped reads) [19] and annotated with the algorithm Basic Local Alignment Search Tools (BLAST) (2.2.23). Plots of the genes of interest expression patterns were made, as described below.

### 2.7. Flow Cytometry

Flow cytometry was performed in cells isolated from fresh milk samples by either staining immediately (*n* = 11) or fixed in 1% paraformaldehyde 2/3% sucrose in PBS for subsequent staining the following day (*n* = 4). When immediately stained, 2 million cells were separated into Eppendorf tubes. Conjugated extracellular antibodies were added to cells (Table S2) in 100 μL of 2% foetal bovine serum (Fisher Biotec, Wembley, WA, Australia) PBS and incubated for 30 min at 4 °C shielded from light. When immediately fixed, the cells were stained the next day with antibodies against membrane proteins (Table S2), diluted in PBS for 30 min on ice and in the dark. All stained cells were then washed twice in PBS (10,000 g for 30 s) and fixed in 3% paraformaldehyde 2/3% sucrose in PBS for 20 min. Subsequently, cells were washed again twice in PBS. While optimizing the technique, it was observed that the antibody against granzyme A worked better when the permeabilization was conducted with 0.05% Tween, therefore antibodies for intracellular staining were diluted in 0.3% saponin or 0.05% Tween in PBS, were added for 30 min, at room temperature, in the dark. Cells were subsequently washed once with 0.1% saponin or 0.05% Tween in PBS (10000 g for 30 s) and then in PBS before resuspending the cells in PBS for subsequent data acquisition. Cells were either analyzed with a BD Accuri C6 plus flow cytometer or in case of triple stainings, cells were measured with a BD FACS Canto. FCS files were then analyzed with the version 10.2 of the software FlowJo™. To avoid doublets, single cells were gated using forward scatter area (FSC-A) versus forward scatter height (FSC-H) (Figure S1). Single cells were further separated in three populations (Figure S1) to examine the presence of granzyme A, granzyme B, granulysin and perforin in immune and epithelial cells.

### 2.8. Statistical Analysis

Statistical analyses were carried out using R 3.3.2. for Mac OSX, with additional packages ggplot2 [20], lattice [21], nlme [22], FactoMineR [23] and factoextra for longitudinal plots, box and whisker plots, mixed linear effects models, principal component analysis (PCA) and plots respectively. Longitudinal spaghetti plots of PS cells, HM cells and RMT gene expression obtained from the sequencing dataset of PRF1, GNLY, GZMA, GZMB, GZMH and GZMM were plotted. Linear mixed effects modeling was used to investigate correlation between qRT-PCR analyzed gene expression products and lactation stage, with participant being a fixed effect. Box and whisker plots were created for gene expression products of all genes resulting from qRT-PCR analysis of HM cells taken from healthy participants. Correlations between expression of the different genes in HM cells taken from healthy participants assessed with qRT-PCR was examined using pairs plots and PCA analysis. Differences in gene expression products assessed with qRT-PCR between healthy and mastitis participants were investigated using PCA and dot plots.

## 3. Results

### 3.1. Analysis of mRNA Encoding Antimicrobial Proteins in Human Milk Cells

#### 3.1.1. Higher Expression of Immune Cell Genes in the Mammary Gland and Milk Cells Taken during Pregnancy and Early Lactation

**Figure 1:**
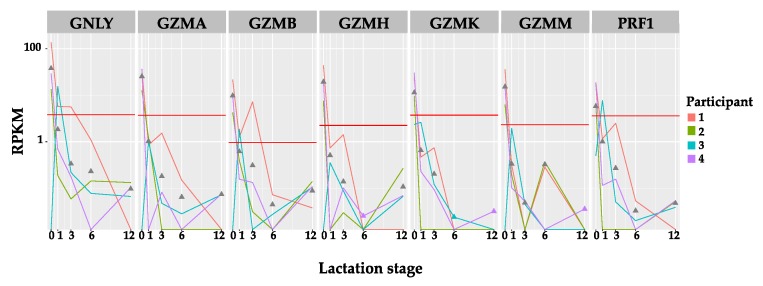
Expression of the genes encoding granzyme A (GZMA), granzyme B (GZMB), granzyme H (GZMH), granzyme M (GZMM), granulysin (GNLY) and perforin (PRF1) expressed in Reads Per Kilobase per Million mapped reads (RPKM) in longitudinal human milk cells (coloured lines) and pooled resting mammary tissue (RMT) (straight red line). Four lactating participants provided pre-partum secretion (PS) cells and four subsequent human milk cell samples at months 1, 3, 6 and 12 of lactation. Average expression at each time point is represented by grey triangles. In general, there is a decrease over lactation period with the highest expression in the pre-partum secretions. Moreover, PS cells show higher gene expression for all immune proteins in comparison with RMT.

Analysis of a prior human mammary transcriptome dataset (GEO Series accession number GSE85494) for immune related genes perforin (PRF1), granulysin (GNLY), granzyme A, B, H and M (GZMA, GZMB, GZMH, GZMM) found the highest expression in pre-partum secretions compared with purchased resting mammary tissue (RMT) mRNA (see Section 2, RNA extraction) and human milk (HM) cells (Figure 1). Overall, the average Reads Per Kilobase per Million mapped reads (RPKM) expression of HM cells for these genes were highest in the prepartum secretions, except for PRF1 which had a similar expression in RMT. Interestingly, whilst post-partum the levels of gene expression generally decreased over the course of 12 months, it appeared there was a slight increase in the expression of GZMA, GZMB, GZMH and PRF1 from 6 to 12 months in 1–2 samples.

#### 3.1.2. Expression Level of Immune Protein Encoding Genes Shows Little Association with Time Post-Partum

**Figure 2:**
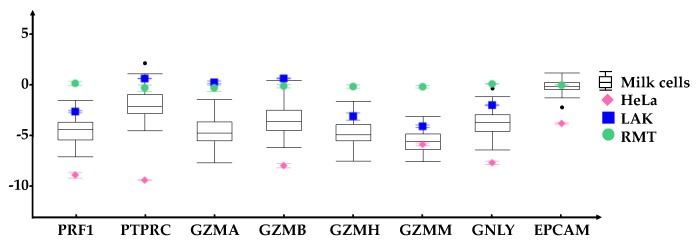
Box and whisker plots of the expression of the immune related genes PRF1, PTPRC, GZMA, GZMB, GZMH, GZMM, GNLY and the epithelial marker EPCAM in 65 HM cell samples (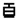). For each gene, the range of expression is shown by the whiskers of the plot and the interquartile ranges are displayed as the upper and lower sections of the boxes. The median expression in milk cells for each gene is represented by the horizontal line going through the box. In the case of milk cell outliers, these are represented by black circles. HeLa cells (◆) were used as a negative control whereas Lymphokine Activated Killer cells (LAK, ■) were a positive control for immune markers. All genes were normalized to resting mammary tissue (RMT, ●). The measured standard error of the mean for these reference samples are represented by error bars. All immune related genes were expressed in the HM cells, although in some cases having a lower expression compared to RMT and LAK.

Expression analysis of the immune cell gene products was expanded in a larger pool of participants (*n* = 24) who provided multiple (1–3) HM cell samples within the first six months of lactation for qRT-PCR analysis (Figure 2). A total of 65 HM cell samples from healthy participants were analysed for PRF1, GZMA, GZMB, GZMH, GZMM, GNLY, the gene encoding the immune cell marker CD45 (PTPRC) and epithelial cell adhesion marker (EPCAM). Many of the HM cell samples expressed all genes, where EPCAM was the most highly expressed gene and GZMM was the least expressed gene (Figure 2). The highest variation of gene expression between participants was found in GZMB (Figure 2, Table S3). Interestingly GZMB expression was found to be negatively associated with lactation stage (*p*-value 0.047, Table S4, Figure S2). GZMA, GZMH, GZMM, GNLY and PTPRC showed no association between expression levels and time post-partum (Table S4, Figure S2) whereas PRF1 had a borderline significant decrease (*p*-value 0.068) (Table S4). Analysis between expression of selected gene products with infant post-partum age revealed inter-individual differences, with participant as an influencing factor on the levels of gene expression.

#### 3.1.3. Negative Association between EPCAM and Immune Markers

**Figure 3:**
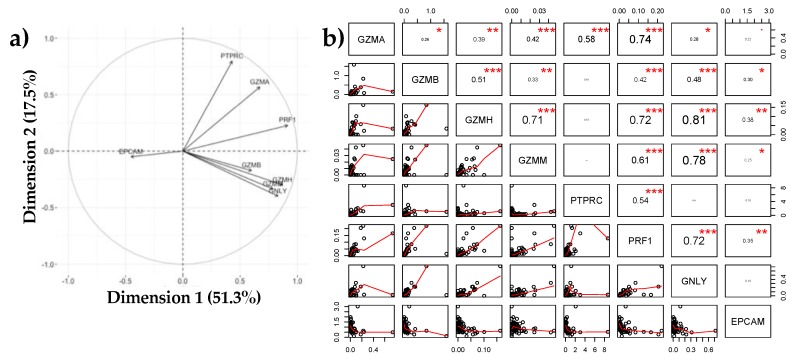
Associations between immune (GZMA, GZMB, GZMH, GZMM, GNLY, PRF1, PTPRC) and epithelial cell markers (EPCAM) analysed with (**a**) principal component analysis (PCA) and (**b**) Linear modelling. Half of the variation could be explained by difference between EPCAM and the immune markers. Moreover, 17.5% was due to the difference between expression of genes encoding granzyme B (GZMB), granzyme H (GZMH), granzyme M (GZMM) and granulysin (GNLY) compared to granzyme A (GZMA), perforin (PRF1) and immune cell marker PTPRC. Linear associations displayed a positive association of gene expression between PRF1 and GZMA, PRF1 and GZMB, PRF1 and PTPRC as well as between GZMA and GZMB. On the other hand a negative association exists between expression of EPCAM and GNLY. *** represents highly correlative genes (*r*^2^ > 0.5), ** moderately correlative (0.5 > *r*^2^ > 0.3) and * low correlation between genes (*r*^2^ < 0.3).

Principal component analysis (PCA) revealed a negative association between expression of EPCAM and the immune cell related gene products PTPRC, GZMA, GZMB, GZMH, GZMM, GNLY and PRF1 (Figure 3) with 51.3% of total variation explained by the differences in these genes (Figure 3a). Linear analysis of the markers (Figure 3b) showed an association between mRNA levels of PRF1 with GZMA (*r*^2^ = 0.74), GZMH (*r*^2^ = 0.72), GZMM (*r*^2^ = 0.61), PTPRC (*r*^2^ = 0.54) or GNLY (*r*^2^ = 0.72). Furthermore, associations between expression of PTPRC and GZMA (*r*^2^ = 0.58) as well as GNLY with GZMH (*r*^2^ = 0.81) or GZMM (*r*^2^ = 0.78) were found. There also appears to be a correlation between expression of GZMH with GZMM (*r*^2^ = 0.71) or GZMB (*r*^2^ = 0.51) (Figure 3b). In contrast, there is a negative association between expression of EPCAM and PTPRC (*r*^2^ = 0.18), GNLY (*r*^2^ = 0.19) and GZMA (*r*^2^ = 0.22) (Figure 3b).

#### 3.1.4. Participants with Mastitis Show a Higher Expression of Immune Genes Compared to Healthy Participants

**Figure 4:**
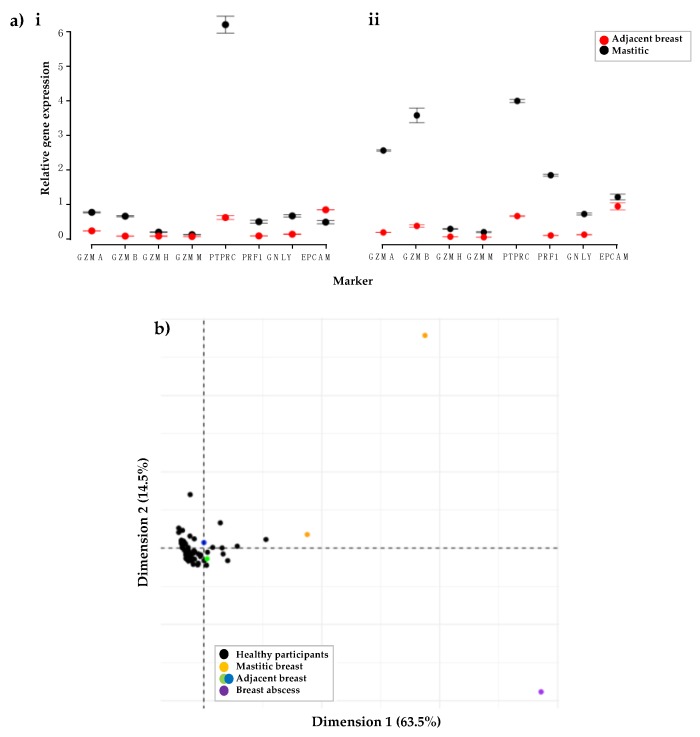
Comparisons of gene expression of immune genes in HM cells taken under different conditions. (**a**) Compares gene expression of HM cells taken from two participants (**i** and **ii**) with breasts affected by mastitis compared to the adjacent breast. PTPRC gene expression was upregulated in both participants (**i** and **ii**), whereas GZMA, GZMB and PRF1 was higher only in the second participant. Error bars represent the calculated standard error of the mean. (**b**) Principal component analysis (PCA) of the variance found between immune related gene expression in human milk cells taken from healthy participants compared to those from participants with mastitis or a breast abscess.

Three participants with mastitis (one with an abscess) provided HM cell samples from the affected breast. Moreover, two of the participants provided an additional sample from the adjacent healthy breast. Compared to the healthy breast, the two mastitis samples from the infected breast displayed a higher expression of immune cell related genes (Figure 4). There was much higher expression of PTPRC in the mastitis sample compared to the adjacent breast in both participants (Figure 4). In addition, the expression of GZMA, GZMB and PRF1 was increased in the mastitis sample of one participant when compared to the adjacent breast (Figure 4a(i)). Principal component analysis (PCA) of gene expression of all healthy and mastitic HM cell samples revealed great variation between healthy participants and the mastitis or breast abscess samples (Figure 4b).

### 3.2. Analysis of Antimicrobial Proteins in Human Milk Cells

#### 3.2.1. Expression at the Protein Level of All Immune Proteins in Healthy Participants

**Figure 5:**
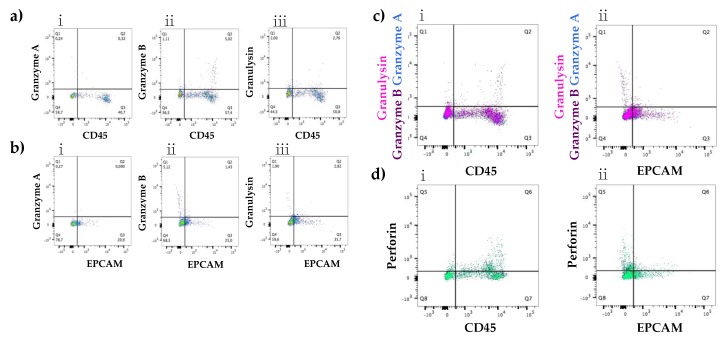
Co-expression of immune proteins granulysin, granzyme A, granzyme B and perforin with immune cell marker CD45 or epithelial marker EPCAM. Granzyme A, granzyme B and granulysin were all co-expressed with CD45 (0.3%, 5.0% and 2.8% respectively) positive cells (**a**(**i**–**iii**)), whereas little co-expression was found between the immune proteins and the epithelial cell marker EPCAM (0.1%, 1.4% and 2.8% respectively) (**b**(**i**–**iii**)). Superposition of the co-expression of granulysin, granzyme B and granzyme A in CD45^+^ (**c**(**i**)) and EPCAM^+^ (**c**(**ii**)). Perforin co-expression with CD45^+^ (**d**(**i**)) cells at a level of 23.7% whereas only 5.2% with EPCAM^+^ (**d**(**ii**)) cells.

Flow cytometry was conducted to investigate the presence of the immune proteins in a healthy lactating population with infants under three months post-partum. Single cells were gated in each sample before separation into three cell populations (Figure S1). Both epithelial (EPCAM) and immune cell (CD45) markers were expressed in all samples with a median of 4.5% and 7.8% respectively, in the total cell population (Table 2). The presence of granulysin and perforin was also confirmed in all samples with a median of 1.6% and 1.4% of cells (Table 2), respectively. Granzyme A was not found in one out of eight samples, whereas granzyme B was not found in two out of twelve samples. The median percentage of cells of healthy participants expressing granzyme A was 0.3% (Table 2). When the sample not expressing granzyme A was taken out, the median was still similar with 0.4% of cells expressing the immune protein granzyme A. In contrast, the median for granzyme B expression in all samples was 2.7% (Table 2) whereas when the samples not expressing granzyme B were taken out, the median increased to 3.01%. When looking at the different cell populations, a high presence of CD45 positive cells (51.5%) exists in cell population 1 (Table 2). Moreover this population included a large cell population expressing granzyme B (11.9%), granulysin (11.7%) and perforin (8.4%) positive cells (Table 3). Epithelial cell marker EPCAM was present in all three gated populations at a similar level (population 1: 18.2%, population 2: 20.2% and population 3: 19.9%, Table 2). All immune related proteins were more highly expressed in CD45 positive cells in comparison to EPCAM positive cells (Table 3), as shown for granzyme B (CD45: 5.2%, EPCAM: 0.5%) (Figure 5). Moreover, there seem to be a co-localization between granzyme B, granulysin and perforin with the highest percentage in population 1 (Table S5).

#### 3.2.2. Increased Number of Immune Cells and Higher Expression of Immune Proteins in Mastitic Milk Samples in Comparison to Healthy Milk Samples

**Figure 6:**
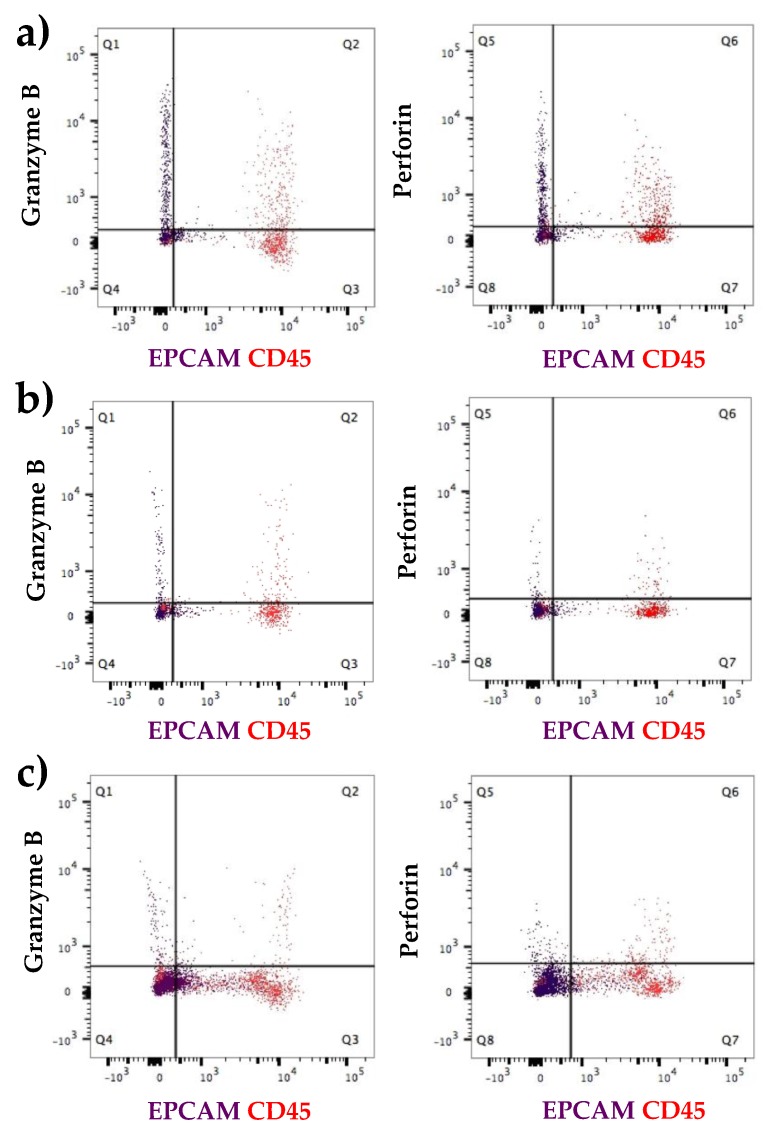
Flow cytometry analysis of human milk cells taken from a participant suffering from (**a**) mastitis with comparison sample of the (**b**) adjacent breast of the same participant and (**c**) compared to a healthy participant. Increased numbers of CD45 positive cells expressing of granzyme B and perforin, whereas no co-expression observed in EPCAM positive cells. Higher expression of immune proteins in CD45 cells was evident in the mastitis affected participant when compared to healthy participants.

HM cells taken from a single participant with mastitis from both the affected and tender adjacent breast were examined for protein expression of immune, antimicrobial and epithelial markers and compared to results from the healthy population (Table 2 and Table 3, Figure 6). Cells taken from the mastitic breast were stained for the markers CD45, granzyme B, perforin and EPCAM due to the low cell number. CD45 positive cells were in a higher proportion in the mastitic sample (12.2% of the whole cell population, Table 2) compared to 8.4% and 7.8% in the adjacent breast and healthy population respectively (Table 2). Similarly, population 1 in healthy participant’s milk showed the highest expression of immune components (Table 2). In this particular population (population 1), the mastitis sample contained a significantly higher number of CD45 positive cells (78.2%) and higher amount of granzyme B positive cells (37.7%) in comparison with the sample from the adjacent breast (64.8% CD45^+^, 17.4% granzyme B^+^) or healthy population (51.5% CD45^+^, 11.9% granzyme B^+^) (Table 2). It also appeared that the sample from the adjacent breast contained slightly higher amounts of CD45 positive cells as well as cells expressing the immune proteins granzyme A and B in comparison to the cohort of healthy population (*n* = 12) (Table 2). Colocalization of granzyme B and perforin in CD45 and EPCAM positive cells from population 1 is shown in Figure 6. In the mastitis sample, clear co-expression of granzyme B in CD45 cells (27.0%, Table 3) as well as perforin in CD45 cells (34.1%, Table 3) is evident. Interestingly, there was also expression of the immune proteins granzyme B (2.2%, Table 3) and perforin (2.1%, Table 3) in cells expressing the epithelial marker EPCAM.

#### 3.2.3. Participant Recovering from Non-Mammary Surgery Shows Higher Expression of Immune Proteins Compared to Healthy Participants

**Figure 7:**
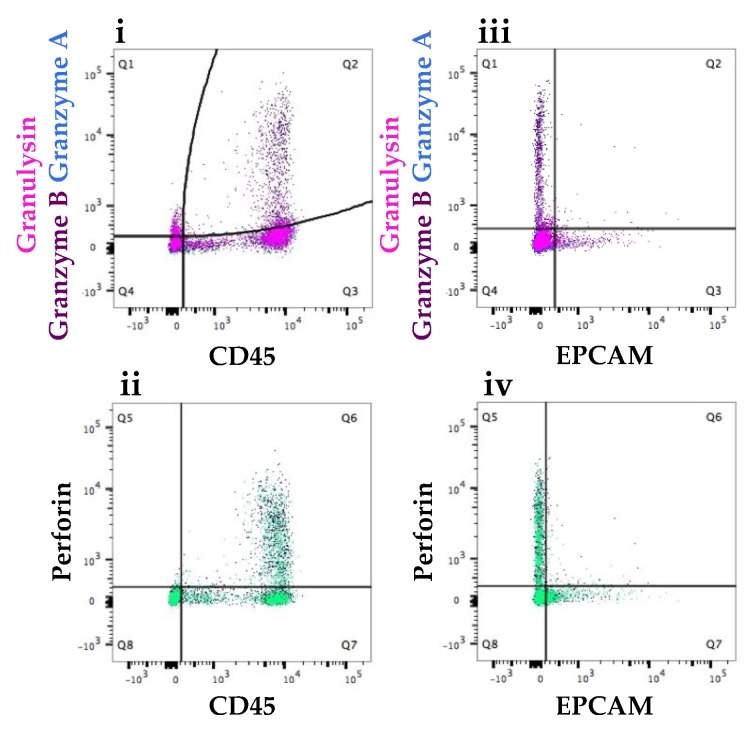
Expression of granzyme A, granyme B, granulysin and perforin in CD45 positive cells and EPCAM positive cells in population 1 in a participant recovering from a non-breast related surgery. Dot plots revealed increased number of CD45^+^ cells (**i**,**ii**) expressing the immune proteins compared with EPCAM^+^ cells (**iii**,**iv**). Expression of the immune proteins in the EPCAM positive cells was low and similar in both participant populations.

HM cells (84 days post-partum) isolated from a participant recovering from a reparative surgery of the hand after a household accident, showed a higher expression of antimicrobial proteins (Figure 7) compared to the median expression of healthy HM cells (Table 2) indicative of heightened levels of activated or memory t-cells. Higher levels of granzyme B, granulysin and perforin were observed in HM cells from the participant that had undergone non-mammary surgery (Figure 7, Table 2). Curved gating was chosen for the double staining of CD45 and granzyme A, CD45 and granzyme B as well as CD45 and granulysin to better exclude the false positive measurements (Figure 7). CD45 positive cells expressed granzyme B (4.4%) as well as granulysin (4.1%) (Table 3). Similar to the mastitis sample, the presence of these immune proteins in EPCAM positive cells was minor. The same tendency is observed with perforin, where the protein was found in higher amounts in CD45 positive cells (23.7%) compared to EPCAM positive cells (5.2%) (Table 3).

## 4. Discussion

Human milk is known to contain immune cells [7] and display antimicrobial properties [24], little is known about the purpose of the cells in the milk or the mechanisms involved in the antibacterial activity. To better understand the actions of immune cells in milk, this study examined the presence of the known antimicrobial proteins perforin, granzymes and granulysin in HM. This study confirms for the first time the expression of the genes GZMA, GZMB, GZMH, GZMM, GNLY and PRF1 in resting mammary tissue, prepartum secretion cells and human milk cells taken from a larger population of women at different stages of lactation. All HM cell samples within this study contained cells positive for the protein CD45 that co-stained with granulysin and perforin and in most cases for granzyme A and B. Whilst EPCAM positive cells were also identified in all samples, co-staining with the investigated immune proteins was minimal. Further investigation revealed that HM cells from participants with local or systemic inflammation had a higher protein expression of CD45 positive cells compared with healthy participants, which is consistent with data from previous studies [8]. The presence and variation of immunological factors in human milk cells in both healthy and participants with inflammation suggests a selected prevalence of activated immune T-cells in HM that also expresses the cytotoxic immune proteins, likely involved both in maternal mammary gland and overall infant protection.

Extending previous studies examining immune cells in human milk, antimicrobial proteins not previously identified in milk cells were found and varied depending on the stage of mammary gland maturation. Investigation of the immune cell related genes identified in mammary transcriptomic data revealed a similar expression pattern between milk leukocyte content and lactation stage with previous data, showing a decreased immune cell content over lactation period [3,7,8]. Pre-partum cells, resting mammary tissue extracts and milk cells at month 1 and 3 had the highest levels of perforin, granulysin and granzymes whilst lower levels were observed in milk samples of the later months. Despite this, gene expression analysis using qRT-PCR did not find a linear relationship between immune gene expression and lactation stage over the first four months except for GZMB where a significant decrease over lactation stage was found. This may reflect the elevated levels of immune cells naturally present in milk from the first 4 months of lactation, which was specifically examined in the qRT-PCR experiments or may suggest that immune protein content is different between women despite infant age in early lactation. It was found that participant was an influencing factor on antimicrobial protein expression but further investigation should consider a larger cohort of women with samples examined at the same lactation stage. 

According to previous studies [3,25,26], different populations of T lymphocytes are present in the milk compared to the peripheral blood circulation. Associations between the expression of PRF1 and the genes encoding for granzymes and granulysin (Figure 3) in HM cells supports previous findings identifying increased expression of effector and memory T-cells in HM in comparison to peripheral blood [25,27], as these proteins are only present after activation of the lymphocytes [28]. Further investigations should include specific antibodies against activated T lymphocytes markers such as CD45RO+ or HLA-DR [29]. A linear association analysis of the immune related gene products showed a positive correlation between PRF1 and the genes encoding for granzymes and granulysin. This observation could mean that the expression of these proteins might be linked, possibly by the same expression control mechanisms. Co-expression of perforin with granulysin and granzymes in CD45 positive HM cells found with flow cytometry also confirms this theory as the efficiency of the proteins is higher when all three proteins are working together [11,12]. Surprisingly, some co-expression was found between EPCAM and the immune proteins (Figure 6) which may indicate low level expression of immune proteins in epithelial cells, epithelial cell uptake of exocytosed cytotoxic granules from activated leukocytes [28,30] or low levels of cell aggregates in the FACS data. Future studies should further investigate the presence of immunological proteins in epithelial cells or whether granules containing immune proteins are released into the milk in cases of infection. Results from this study suggest that leukocytes in the milk are increased not only in the case of mammary inflammation but also in the case of systemic inflammation.

Investigated immune components (cells and immune proteins) were not only more prevalent in HM taken from the breast affected by mastitis but also in the adjacent breast. Interestingly immune components were also elevated in a participant who had undergone non-mammary surgery, compared to HM cells from healthy participants. Mastitis, being an infection of the breast tissue [8], creates a local inflammation and a systemic response leading to an increase of circulating immune cells in the blood [31]. Consequently, a higher quantity of lymphocytes infiltrating mammary tissue likely being the cause of heightened immune cells in the milk during mastitis [8]. As shown by flow cytometry, there were a heightened number of CD45 positive HM cells with co-expression of granzymes, granulysin and perforin from a participant with mastitis (Figure 6). As mastitis is usually a bacterial infection, the increased presence of immune cells and increased expression of antimicrobial proteins having an antibacterial effect was an expected outcome. In addition, elevated levels of CD45 cells with co-expression of the investigated immune proteins was also found in the milk obtained from the non-mastitic adjacent breast, although they were at much lower levels (Figure 6). The presence of immune related proteins in healthy participants, may suggest that they might not only play a role in the fight against an infection, but also in the prevention of one. This indicates that immune cells may not only have a role in the development of the immune system of the infant [32], but also in the protection of the lactating breast [3]. Follow-up studies should consider including complimentary blood samples alongside with milk to have an appropriate comparison point between milk and blood leukocytes.

## 5. Conclusions

This study showed for the first time the expression of the antimicrobial proteins perforin, granulysin and different granzymes at the protein and mRNA level in HM cells, RMT and PS cells. Furthermore, it provided confirmation that HM from healthy women is enriched in cells that carry hallmarks of activated or memory T-cells, which are elevated in case of maternal infection. Presence of these cells may indicate a purpose in the protection of the vulnerable infant or as a mechanism to defend the maternal breast against infection however further investigations should done to clarify this.

## Figures and Tables

**Table 1:** Demographics of study participants engaged in the study.

	Median (Range)		
	All Participants	PCR Participants	Flow Cytometry Participants
Maternal characteristics	*n* = 36	*n* = 24	*n* = 13
Age (years)	34 (27–45)	33 (27–45)	35.5 (34–38)
Body Mass Index (BMI)	24 (19.8–31.9)	22.5 (19.8–27.9)	25.6 (22–31.9)
Parity	2 (1–4)	2 (1–4)	2 (1–3)
Infant characteristics	*n* = 36	*n* = 24	*n* = 13
Gestational age (days)	278 (249–301)	278 (249–301)	275 (252–281)
Infant age at collection (days)	47 (4–142)	45 (4–142)	57 (37–84)
Milk characteristics	*n* = 85	*n* = 70	*n* = 15
Volume of milk (mL)	50 (0.61–490)	50 (0.61–490)	62 (35–195)
Total cell count (cells/mL)	16.4 (1.9–214.5)	16.55 (1.9–214.5)	13.8 (4.9–63.8)
Viability (%)	97.4 (53.2–100)	98.1 (53.2–100)	93.6 (84.7–96.5)

**Table 2:** Flow cytometric analysis of immune and epithelial cell proteins in HM cells (%) taken from healthy, mastitis and non-mammary post-surgery participants. Single marker expression.

	Healthy (*n* = 12)	Mastitis Sample (*n* = 1)		Non-Mammary Surgery Sample (*n* = 1)
	Median (Range)	Mastitis Breast	Non-Mastitic Adjacent Breast	
CD45^+^ (%)				
All single cells	7.8 (2.1–13.4)	12.2	8.4	26.7
Population 1	51.5 (1.5–66.7)	78.2	64.8	66.8
Population 2	11.8 (1.9–51.5)	69.6	73.5	71.8
Population 3	2.7 (1.2–8.7)	3.2	1.5	17.3
Granzyme A^+^ (%)				
All single cells	0.3 (0.0–10.6)		0.8	0
Population 1	1.9 (0.4–82.0)		7.1	4.3
Population 2	0.5 (0.0–19.2)		1.6	0
Population 3	0.9 (0.00–1.3)		0.4	0
Granzyme B^+^ (%)				
All single cells	2.7 (0.0–4.1)	0.4	1.2	2.6
Population 1	11.9 (0.3–39.6)	37.7	17.4	33.1
Population 2	0.8 (0.0–9.5)	1.1	1.7	7.8
Population 3	0.4 (0.0–4.3)	0.3	0.6	10.1
Granulysin^+^ (%)				
All single cells	1.6 (0.3–12.3)		1.6	2.2
Population 1	11.7 (1.2–18.8)		4.3	13
Population 2	2.0 (0.4–12.8)		4.5	12.4
Population 3	15.5 (0.6–30.3)		1.1	15.7
Perforin^+^ (%)				
All single cells	1.4 (0.5–2.5)	2.3	1.4	2.4
Population 1	8.4 (0.0–31.7)	37.4	8.6	27
Population 2	2.2 (0.5–13.9)	4.5	9.7	10.3
Population 3	1.3 (0.8–9.5)	0.3	0.6	1.1
EPCAM^+^ (%)				
All single cells	4.5 (3.8–12.8)	6.6	5.1	3.4
Population 1	18.2 (3.5–36.8)	18.2	11.4	13.4
Population 2	20.2 (5.8–26.0)	23.4	12.9	9.5
Population 3	19.9 (9.4–30.3)	19.3	16.5	7.1

**Table 3:** Flow cytometric analysis of immune and epithelial cell proteins in HM cells (%) taken from healthy, mastitis and non-mammary post-surgery participants. Double stainings between either CD45 or EPCAM positive cells and immune proteins.

	Healthy (*n* = 12)	Mastitis Sample (*n* = 1)		Non-Mammary Surgery Sample (*n* = 1)
	Median (Range)	Mastitis Breast	Adjacent Breast	
CD45–Granzyme A (%)				
All single cells	0.2 (0.0–14.9)		0.2	0.4
Population 1	1.0 (0.3–32.6)		10.9	5.4
Population 2	0.2 (0.0–50.0)		3.5	1.3
Population 3	0.2 (0.0–0.4)		0.2	0
CD45–Granzyme B (%)				
All single cells	0.7 (0.0–5.5)	0.5	0.4	4.4
Population 1	5.2 (0.0–26.8)	27	17.2	33.4
Population 2	0.3 (0.0–13.9)	0.3	4.2	20.3
Population 3	0.0 (0.0–0.3)	0	0.3	1.4
CD45–Granulysin (%)				
All single cells	0.7 (0.0–10.4)		0.3	4.1
Population 1	3.2 (0.8–21.1)		4.4	12.9
Population 2	3.6 (0.0–13.4)		5.8	34
Population 3	13.6 (0.2–27.1)		0.1	0.9
CD45–Perforin (%)				
All single cells	0.9 (0.6–8.3)	0.8	0.9	23.7
Population 1	11.0 (0.1–35.2)	34.1	8.2	27.7
Population 2	2.1 (0.1–26.8)	3.9	10.6	48.2
Population 3	0.7 (0.1–1.8)	0.7	0.1	4.8
EPCAM–Granzyme A (%)				
All single cells	0.0 (0.0–0.2)		0.1	0
Population 1	0.1 (0.0–35.2)		0.1	0.1
Population 2	0.4 (0.0–1.5)		0.6	0
Population 3	1.0 (0.2–1.8)		0.2	0
PCAM–Granzyme B (%)				
All single cells	0.2 (0.1–1.0)	1.3	0	1.5
Population 1	0.5 (0.3–3.5)	2.2	0.6	0.9
Population 2	1.4 (0.0–3.0)	0.3	0.5	6.1
Population 3	1.3 (0.2–2.3)	0	0.2	1.1
EPCAM–Granulysin (%)				
All single cells	0.2 (0.0–2.1)		0.1	2.4
Population 1	1.5 (0.2–3.1)		1.8	1
Population 2	0.6 (0.1–1.4)		1.4	7.9
Population 3	2.6 (0.2–5.0)		0.1	1.9
EPCAM–Perforin (%)				
All single cells	0.2 (0.0–5.5)	0.2	0.1	5.2
Population 1	3.3 (1.1–9.5)	2.1	0.5	2.2
Population 2	1.7 (0.3–8.4)	0.7	1.1	7.4
Population 3	0.4 (0.3–0.4)	0.3	0	1.8

## Supplementary Materials

The following are available online at http://www.mdpi.com/2072-6643/10/9/1230/s1, Figure S1: Gating around single cells using forward scatter area (FSC-A) and forward scatter height (FSC-H). Three populations were then gated using only single cells. Figure S2: Relative quantitation (RQ) of the expression of the epithelial marker EPCAM, the immune cell marker PTPRC and the genes coding for granzyme A (GZMA), granzyme B (GZMB), granzyme H (GZMH), granzyme M (GZMM), granulysin (GNLY) and perforin (PRF1) distributed according to the time period post-partum. Table S1: Taqman probes from Life Technologies. Table S2: Antibodies used for FACS analysis. Table S3: Expression of selected genes in human milk cells (HMC) measured via RT-PCR, normalised to either lymphokine activated killer cells (LAK) or resting tissue (RT). Table S4: Univariate linear mixed modelling of days post-partum and antimicrobial protein genes, with participant as an influencing factor on gene expression. Table S5: Flow cytometric analysis of immune and epithelial cell proteins in HM cells (%) taken from healthy, mastitis and non-related surgical patient participants.
