# Supplementary material for: Expression of Granulisyn, Perforin and Granzymes in Human Milk over Lactation and in the Case of Maternal Infection
Source: Nutrients. 2018 Sep 4;10(9):1230. doi: 10.3390/nu10091230 (PMC6163887; doi:10.3390/nu10091230)
Supplement: Supplementary file 1 [file nutrients-10-01230-s001.docx]

***Supplementary information***

**Antimicrobial Proteins in Human Milk Cells**

**Alecia-Jane Twigger^1†*^, Gwendoline K. Küffer^2†^, Donna T. Geddes^3^, Luis Filgueria^2^**

**Figure S1:** Gating around single cells using forward scatter area (FSC-A) and forward scatter height (FSC-H). Population 1 (P1), population 2 (P2) and population 3 (P3) were then gated using only single cells.

**Figure S2:** Relative quantitation (RQ) of the expression of the epithelial marker EPCAM, the immune cell marker PTPRC and the genes coding for granzyme A (GZMA), granzyme B (GZMB), granzyme H (GZMH), granzyme M (GZMM), granulysin (GNLY) and perforin (PRF1) distributed according to the time period post-partum. Individual participants are represented with different coloured line plots

**Table S1:** Taqman probes from Life Technologies

| **Gene** | **Catalogue number** |
| --- | --- |
| HuGAPDH | HS03929097_g1 |
| PRF1 | Hs00169473_m1 |
| PTPRC | Hs04189704_m1 |
| GZMA | Hs00989184_m1 |
| GZMB | Hs00188051_m1 |
| GZMH | Hs00277212_m1 |
| GZMM | Hs00193417_m1 |
| GNLY | Hs00246266_m1 |
| EPCAM | Hs00158980_m1 |

**Table S2**: Antibodies used for FACS analysis

| Antibody | Dilution | Marker style | Company | Catalogue number |
| --- | --- | --- | --- | --- |
| CD45 – PE-Cy5 | 1:50 | Surface marker | BD Biosciences |  |
| CD45 – FITC | 1:5 | Surface marker | BD Biosciences | 555482 |
| EPCAM - FITC | 1:10 | Surface marker | MACS Miltenyi Biotec | 347197 |
| Prf1 – AF647 | 1:50 | Intracellular marker | BD Biosciences | 563576 |
| GzmA - PE | 1:50 | Intracellular marker | MACS Miltenyi Biotec | 130-099-296 |
| GzmB – PE | 1:10 | Intracellular marker | BD Biosciences | 561142 |
| GNLY – PE | 1:20 | Intracellular marker | BioLegend | 348004 |

**Table S3**: Expression of selected genes in human milk cells (HMC) measured via RT-PCR, normalised to either lymphokine activated killer cells (LAK) or resting tissue (RT). Values within this table represent a relative quantitation (RQ) compared to these reference samples indicating the fold difference in the expression between the sample and the positive control. If the gene expression of the HMC is the same as that of the control, the expression will be equal to 1. Values have been rounded to 3 decimal places and where no expression was found, this is indicated by ‘-‘.

| **Gene** | **Expression in HMC**  **Mean (range)** | **Expression in HeLa** | **Expression in LAK** | **Expression in RT** |
| --- | --- | --- | --- | --- |
| PRF1 | 0.028 (0.001-0.221) | 0.000 | 0.069 | 1.095 |
| PTPRC | 0.511 (0.011-8.679) | 0.000 | 1.756 | 0.711 |
| GZMA | 0.045 (0.000-0.736) | - | 1.211 | 0.705 |
| GZMB | 0.105 (0.002-1.594) | 0.000 | 1.777 | 0.842 |
| GZMH | 0.023 (0.001-0.202) | - | 0.044 | 0.801 |
| GZMM | 0.007 (0.001-0.046) | 0.003 | 0.016 | 0.784 |
| GNLY | 0.080 (0.002-1.240) | 0.000 | 0.130 | 1.037 |
| EPCAM | 1.040 (0.112-3.364) | 0.022 | - | 0.911 |

**Table S4**: Univariate linear mixed modelling of days post-partum and antimicrobial peptide genes, with participant as an influencing factor on gene expression

| **Response Variable** | **Intercept** | **Slope (days post-partum)** | **SE (slope)** | **P-value (slope)** |
| --- | --- | --- | --- | --- |
| GZMA | 0.0611 | -0.000376 | 0.000296 | 0.212 |
| GZMB | 0.169 | -0.00132 | 0.000643 | 0.047 |
| GZMH | 0.0249 | -0.00012 | 0.0000763 | 0.122 |
| GZMM | 0.00813 | -0.0000383 | 0.0000261 | 0.150 |
| GNLY | 0.0857 | -0.000496 | 0.000297 | 0.102 |
| PRF1 | 0.0408 | -0.000241 | 0.000129 | 0.068 |
| PTPRC | 0.739 | -0.00398 | 0.00335 | 0.242 |

**Table S5**: Flow cytometric analysis of immune and epithelial cell proteins in HM cells (%) taken from healthy, mastitis and non-related surgical patient participants. Double stainings between perforin and other immune proteins.

|  | **Healthy (n=12)** | **Mastitis sample (n=1)** | | **Post surgery sample** |
| --- | --- | --- | --- | --- |
|  | Median (Range) | Mastitis breast | Adjacent breast | **(n=1)** |
| Granzyme A – Perforin  All single cells  Population 1  Population 2  Population 3 | 0.0 (0.0 – 0.7)  0.1 (0.0 – 25.5)  0.0 (0.0 – 1.1)  0.1 (0.0 – 0.2) |  | 0.1  3.6  1.8  0.0 | 0.0  1.6  0.1  0.0 |
| Granzyme B – Perforin  All single cells  Population 1  Population 2  Population 3 | 0.4 (0.0 – 2.8)  5.7 (3.4 – 23.0)  0.8 (0.4 – 10.6)  0.3 (0.2 – 0.4) | 0.2  34.4  2.8  0.1 | 0.1  8.6  1.5  0.1 | 2.5  26.7  13.3  0.8 |
| Granulysin – Perforin  All single cells  Population 1  Population 2  Population 3 | 0.8 (0.0 – 1.5)  4.6 (1.7 – 17.6)  1.6 (0.2 – 2.1)  0.5 (0.4 – 0.6) |  | 0.3  3.2  4.8  0.1 | 2.6  15.5  19.7  0.9 |
